# Argon and Oxygen Gas Flow Rate Dependency of Sputtering-Based Indium-Gallium-Zinc Oxide Thin-Film Transistors

**DOI:** 10.3390/mi14071394

**Published:** 2023-07-08

**Authors:** Youngmin Han, Dong Hyun Lee, Eou-Sik Cho, Sang Jik Kwon, Hocheon Yoo

**Affiliations:** Department of Electronic Engineering, Gachon University, Seongnam 13120, Republic of Korea

**Keywords:** a-IGZO, magnetron sputtering, thin-film transistors, oxygen vacancy, oxygen flow rate

## Abstract

Oxygen vacancies are a major factor that controls the electrical characteristics of the amorphous indium-gallium-zinc oxide transistor (a-IGZO TFT). Oxygen vacancies are affected by the composition ratio of the a-IGZO target and the injected oxygen flow rate. In this study, we fabricated three types of a-IGZO TFTs with different oxygen flow rates and then investigated changes in electrical characteristics. Atomic force microscopy (AFM) was performed to analyze the surface morphology of the a-IGZO films according to the oxygen gas rate. Furthermore, X-ray photoelectron spectroscopy (XPS) analysis was performed to confirm changes in oxygen vacancies of a-IGZO films. The optimized a-IGZO TFT has enhanced electrical characteristics such as carrier mobility (μ) of 12.3 cm^2^/V·s, on/off ratio of 1.25 × 10^10^ A/A, subthreshold swing (*S.S.*) of 3.7 V/dec, and turn-on voltage (V_to_) of −3 V. As a result, the optimized a-IGZO TFT has improved electrical characteristics with oxygen vacancies having the highest conductivity.

## 1. Introduction

Since 2004, when Hideo Hosono presented an amorphous indium gallium zinc oxide (a-IGZO) and its use in transparent and flexible thin-film TFTs (TFTs), a-IGZO-based TFTs have drawn considerable attention [1,2,3]. In particular, a-IGZO-based TFTs are currently used in the display industry as this device offers high carrier mobility and, accordingly, sufficient driving current density to operate an organic light-emitting diode (OLED) can be made available [4,5,6]. Furthermore, another merit of a-IGZO-based TFTs is large-area deposition with high uniformity [7,8,9]. Due to these merits, many efforts have been made to implement a new concept of electronic devices such as neuromorphic devices [10,11,12], gas sensors [13,14,15], photodetectors [16,17,18], biosensors [19,20,21], and logic circuits [22,23,24].

One of the most frequently used deposition methods for a-IGZO is sputtering. This deposition method provides a facile deposition of thin films, particularly oxides, by means of sputtering from a “target” source to a “substrate” [25,26,27]. As oxygen vacancy concentration in a-IGZO significantly determines the electrical properties of TFTs, specific deposition conditions, including gas flow rate dependency, should be considered [28], and thus, its optimization should be accompanied. 

In addition, annealing processes of metal oxide semiconductors (MOS) are improving carrier mobility [29,30,31]. Oxygen vacancy generated in MOS crystallized through the annealing process are important factors determining conductivity [32,33]. The oxygen vacancy behaves as an electron donor through a fully occupied defect state. Therefore, the conductivity of MOS increases due to the closer Fermi level and conduction band [34,35]. Compared to chemical doping methods and new designs of MOS-based devices, the annealing process provides a simple method and immediate effect for controlling the electrical characteristics of MOS. However, the high annealing temperature of over 600 °C and the additional pre-processing variables reduce the process compatibility of MOS-based devices [36,37,38].

Here, we investigate the a-IGZO TFT process conditions by controlling the oxygen flow rate without an additional annealing process. The effect of oxygen vacancies controlled by the oxygen gas rate was investigated using atomic force microscopy (AFM) and X-ray photoelectron spectroscopy (XPS) analysis. The a-IGZO TFT fabricated under optimized process conditions exhibits excellent electrical characteristics due to increased conductivity with oxygen vacancies. The carrier mobility and on/off ratio of the optimized a-IGZO TFT are 12.3 cm^2^/V·s and 1.25 × 10^10^ A/A, respectively. Also, the subthreshold swing (*S.S.*) of 3.7 V/dec and a V_to_ of −3 V were achieved.

## 2. Materials and Methods

**Figure 1:**
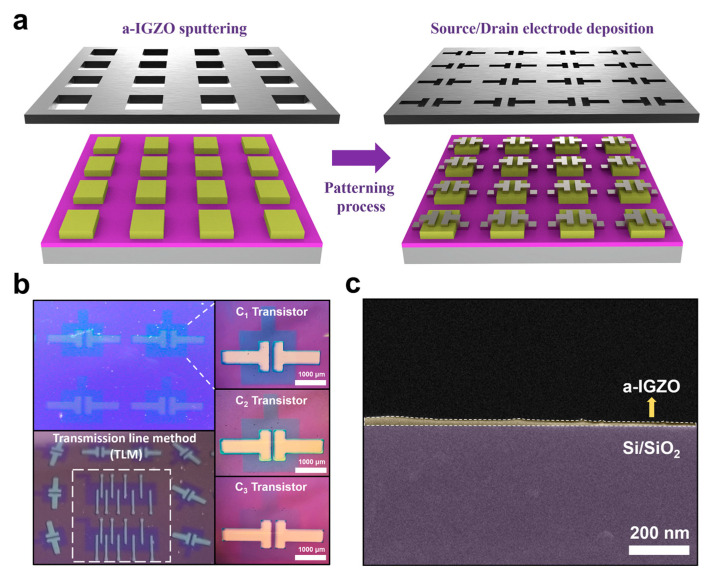
(**a**) 3D schematic of channel and electrode patterning process of a-IGZO TFT using shadow mask. (**b**) Optical microscope image of a-IGZO TFT array according to a-IGZO sputtering conditions. (**c**) Scanning electron microscope image of a-IGZO TFT deposited under optimized process conditions.

To fabricate the a-IGZO TFTs, a heavily boron-doped p-type Si/SiO_2_ (300 nm) was prepared. The SiO_2_ layer was used as the gate dielectric, and a p-type Si layer was applied as the back gate. The Si/SiO_2_ wafer was cleaned with acetone and isopropyl alcohol and then dried with nitrogen gas. a-IGZO (In_2_O_3_: Ga_2_O_3_: ZnO = 1:1:1) for the channel of TFTs was deposited using the radio frequency (RF) magnetron sputtering method. Condition 1 (C_1_) is the deposition method of a-IGZO using only the Ar gas. The gas mixing ratio of O_2_:Ar injected during the deposition of a-IGZO is 1.7:100 (Condition 2, C_2_) and 17:100 (Condition 3, C_3_). The Ti (60 nm) source and drain electrode had deposited electron-beam evaporation. The width (*W*) and length (*L*) of the channels are 100 μm and 1000 μm, respectively. Figure 1a shows the channel and electrode patterning process of a-IGZO TFT using a shadow mask. Figure 1b shows a top-view optical microscope (OM) image of the a-IGZO TFT array. Also, Figure 1c shows the cross-sectional view image of a-IGZO TFT with a scanning electron microscope (SEM), and the thickness of optimized a-IGZO is 8 nm.

The electrical characteristics of a-IGZO TFT were analyzed using a Keithley 4200 (Tektronix, Beaverton, OR, USA) semiconductor parameter analyzer in the air. The electrical characteristics of a-IGZO TFT were investigated according to oxygen gas injection conditions. Atomic force microscopy (AFM) images were measured by XE7 (Park Systems, Suwon, Republic of Korea). X-ray photoelectron spectroscopy (XPS) was measured using AXIS-SUPRA (Kratos, Manchester, UK) at the Korea Basic Science Institute (KBSI). The field-effect mobility and the subthreshold swing (*S.S.*) of TFTs were calculated using the equation:

(1) $$\mu_{electron}=\frac{\partial I_{D}}{\partial V_{G}}\frac{L}{WC_{ox}V_{D}}$$

(2) $$S.S.=\frac{dV_{G}}{d(\log_{10}I_{D})}$$

where *L* and *W* represent the length and width of the channel, and *C* is the capacitance of the gate insulator. The *V_GS_* is the applied gate–source voltage, and *I_D_* is the drain current.

Also, the interface trap density (*D_it_*) of the TFT was derived using the following equation [39]:

(3) $$D_{it}=\frac{C_{ox}}{q^{2}}(\frac{S.S.\;log(e)}{K_{B}T/q}-1)$$

where *q* and *T* represent the elementary electron charge and absolute temperature, respectively. The *K_B_* is the Boltzmann constant, and *e* is the dielectric constant.

## 3. Results

**Figure 2:**
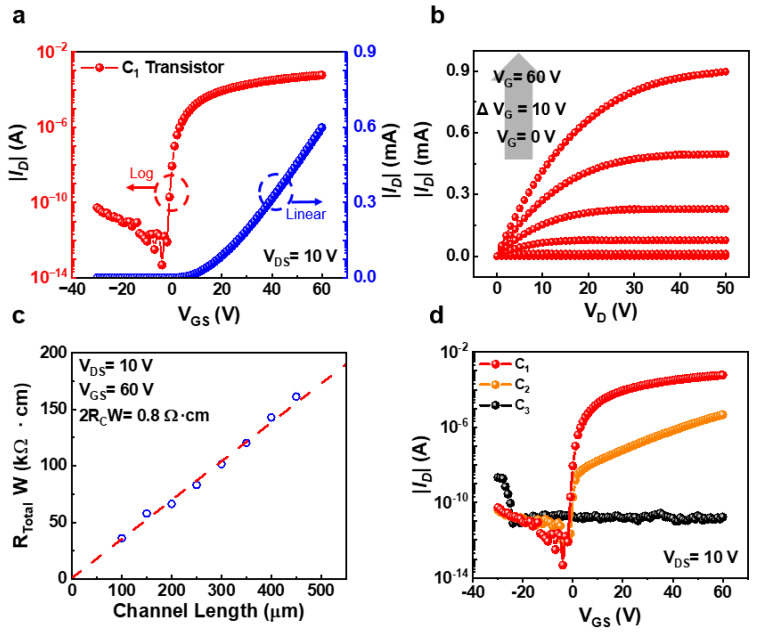
(**a**) Transfer curve of optimized C_1_ TFT. (**b**) Output curve of optimized C_1_ TFT. (**c**) Contact resistance of optimized C_1_ TFT. (**d**) Device electrical characteristics according to oxygen flow variation.

Various variables, such as the atomic composition ratio of the a-IGZO target, O_2_/Ar mixed gas ratio, and thin film thickness, affect the electrical characteristics of a-IGZO TFTs. In particular, oxygen vacancies are essential due to controlling the electrical characteristics of a-IGZO TFTs. Moreover, oxygen vacancies on the surface of a-IGZO are affected by oxygen gas flow rates. For this reason, we fabricated three types of TFTs with different oxygen gas flow rates to investigate changes in electrical characteristics. Figure 2a shows the transfer curve of the C_1_ TFT fabricated under the optimized process conditions. The channel of the C_1_ TFT was deposited without oxygen gas injection. The gate–source voltage of the measured transfer curve was −30 V to 60 V, and a drain voltage of 10 V was applied. The on-current and off-current of the C_1_ TFT were measured to 6 × 10^−4^ A and 4.8 × 10^−14^ A, respectively, and the calculated on/off ratio was measured to as high as 1.25 × 10^10^ A/A. Figure 2b shows the output curve of the C_1_ TFT presenting conventional n-type operation. To measure the output curve, a range of the gate–source voltage is 0 V to 60 V and a drain voltage of 0 V to 50 V were applied, respectively. The contact resistance of a-IGZO TFT was evaluated using the transmission line method (TLM) method. As shown in Figure 2c, the extracted contact resistance is 0.4 Ω·cm using the TLM method. The contact resistance is related to charge injection, which affects electrical characteristics such as mobility and on/off ratio [40,41,42]. Therefore, the reduced contact resistance promotes charge injection to the channel, and the C_1_ TFT had improved mobility and on/off ratio performance. Figure 2d shows the transfer curves with three types of a-IGZO TFTs in different oxygen gas flow rates. The O_2_/Ar mixed gas ratios of the C_2_ and C_3_ TFTs are 1.7:100 and 17:100, respectively. The on-current of the a-IGZO TFT decreases as the oxygen flow rate increases. The electrical characteristics of the optimized a-IGZO TFT depend on the composition ratio of the a-IGZO target. The low conductivity of a-IGZO TFT fabricated with no oxygen component a-IGZO target is overcome by injecting the oxygen gas [43]. On the other hand, the a-IGZO TFT deposited by injecting additional oxygen gas into the a-IGZO target with an oxygen component has reduced oxygen vacancies, resulting in low conductivity. The on-currents at V_GS_ = 60 V of the C_1_ and C_2_ TFTs were measured to 4.7 × 10^−6^ A and 6 × 10^−4^ A, respectively. The C_2_ TFT has 127 times less on-current compared to the C_1_ TFT. Therefore, the on/off ratio of the C_2_ TFT was 1.24 × 10^6^ A/A, which was decreased compared to that of the C_1_ TFT. The C_3_ TFT had reduced oxygen vacancy compared to C_1_ and C_2_ TFTs by injecting the highest amount of oxygen gas during a-IGZO sputtering. The low conductivity of the C_3_ TFT is attributed to the reduced carrier density due to excessive oxygen vacancy. Therefore, the C_3_ TFT has an average current of 67 pA in the gate–source voltage and ranges from −30 V to 60 V and could not be converted to on-state by the positive gate–source bias voltage.

**Figure 3:**
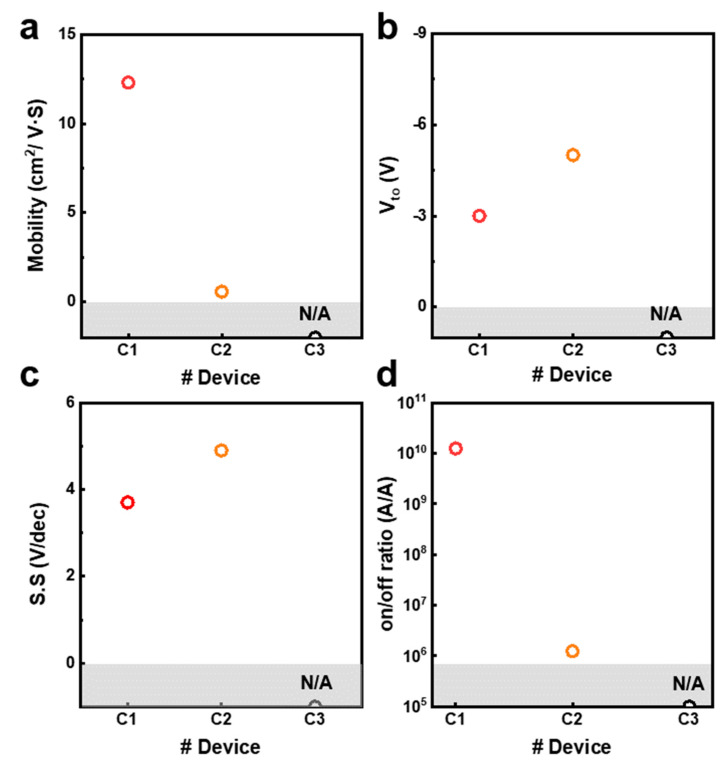
Parameters in three types of a-IGZO TFTs. (**a**) Carrier mobility (μ). (**b**) Turn-on voltage (V_to_). (**c**) Subthreshold swing (S.S). (**d**) On/off ratio.

The electrical parameters with the three types of a-IGZO TFTs were compared (Figure 3a–d). The electrical parameters include carrier mobility (μ), turn-on voltage (V_to_), subthreshold swing (*S.S.*), and on/off ratio. The electrical parameters of the C_3_ TFT were uncalculated due to insufficient switching behavior due to the low conductivity of a-IGZO. The carrier mobility of the C_1_ TFT and C_2_ TFT is 12.3 cm^2^/V·s and 0.58 cm^2^/V·s, respectively. The carrier mobility of the C_1_ TFT is 21 times higher than that of the C_2_ TFT. Also, the V_to_ is −3 V and −5 V, respectively, and *S.S.* is 3.7 V/dec and 4.9 V/dec, respectively. As a result, the optimized C_1_ TFT achieved a high on/off ratio and carrier mobility. In addition, the electrical characteristics of C_1_ transistors with V_to_ close to zero gate–source voltage and low *S.S.* potentially enable low voltage operation and low power consumption. The high electron mobility and reduced *S.S.* of C_1_ TFT compared to C_2_ TFT are achieved due to the reduced oxygen vacancy and interface trap density (*D_it_*_)_ in a-IGZO. The extracted *D_it_* of the C_1_ TFT and C_2_ TFT was 4.4 × 10^12^ cm^−2^ eV^−1^ and 5.8 × 10^12^ cm^−2^ eV^−1^, respectively. Appropriate oxygen vacancy and interface trap density minimize the trapping of charge carriers in C_1_ TFT, thereby increasing charge carrier mobility. Also, they decrease the *S.S.*, inducing clear switching behavior of a-IGZO TFT [44,45,46]. Therefore, optimizing oxygen vacancy and interface trap density is crucial for improving the performance of a-IGZO TFTs, leading to enhanced charge carrier mobility and reduced *S.S.*

**Figure 4:**
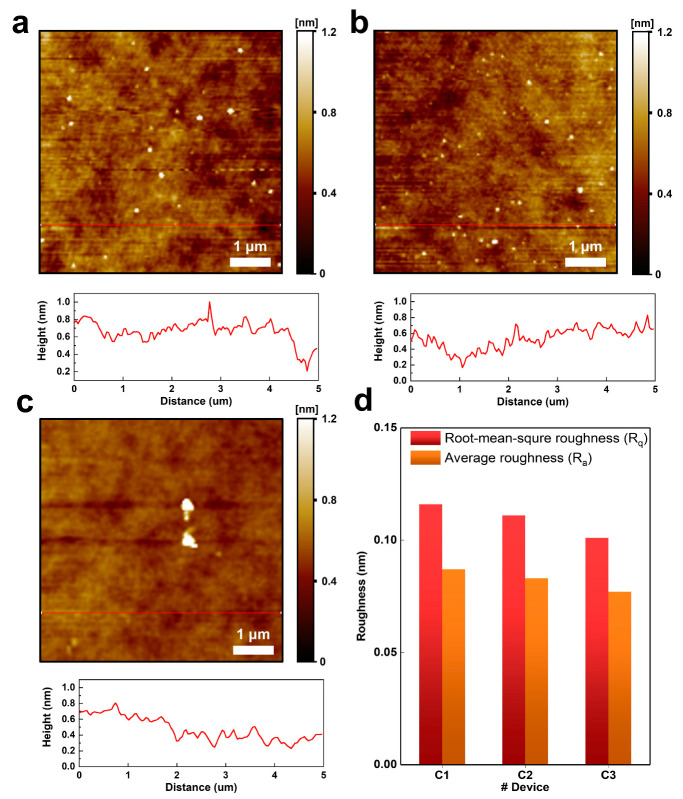
Atomic force microscopy (AFM) image and height information for morphology analysis (**a**) C_1_ TFT. (**b**) C_2_ TFT. (**c**) C_3_ TFT. (**d**) Average roughness (R_a_) and root mean square roughness (R_q_) of three IGZO films with different oxygen flow rates.

Next, we performed an atomic force microscope (AFM) analysis to investigate the morphological characteristics of a-IGZO films fabricated by oxygen flow rate. Figure 4a–c shows the AFM image (5 μM × 5 μM) and height information of a-IGZO films using fabrication methods with C_1_, C_2_, and C_3_, respectively. The height information of the a-IGZO surface did not become rough when oxygen gas flow was increased [47,48]. The morphological characteristics changes on the surface of a-IGZO are related to the concentration of oxygen vacancy. The defect, such as oxygen vacancy, induces rough surface morphology of a-IGZO films. As a result, a high concentration of oxygen vacancy causes the surface of a-IGZO morphology to be rough, while a low concentration of oxygen vacancy induces a smoother surface morphology [31,49]. In addition, the average roughness (R_a_) and root mean square roughness (R_q_) were calculated from the height information of a-IGZO using the AFM measurement (Figure 4d). The R_a_ value of C_1_, C_2_, and C_3_ was 0.134, 0.111, and 0.089 nm, respectively. In addition, the values of R_q_ are 0.151, 0.134, and 0.126 nm, increasing with the oxygen flow rate.

**Figure 5:**
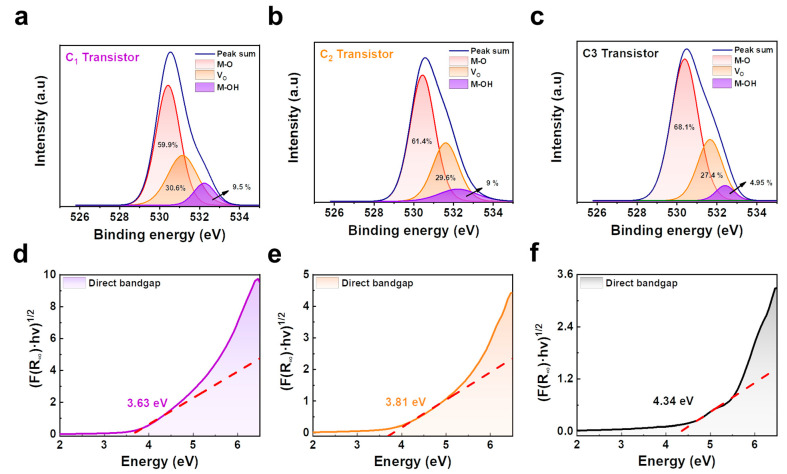
The O 1s peak of XPS spectra in a-IGZO films (**a**) a-IGZO films deposited in C_1_. (**b**) a-IGZO films deposited in C_2_. (**c**) a-IGZO films deposited in C_3_. Optical bandgap of a-IGZO films using Tauc plot method (**d**) a-IGZO films deposited in C_1_. (**e**) a-IGZO films deposited in C_2_. (**f**) a-IGZO films deposited in C_3_.

To investigate the chemical composition difference between the three types of a-IGZO films, X-ray photoelectron spectroscopy (XPS) analysis was performed. Figure 5a–c shows the O 1s spectra of the a-IGZO films according to the oxygen gas flow during deposition. The O 1s spectra of the a-IGZO are separated into sub-peaks associated with O_2_- ions, such as metal-oxide (M-O) bonds, metal-hydroxyl (M-OH) bonds, and oxygen vacancies. The sub-peaks of O 1s indicate binding at 530 eV, 532 eV, and 531 eV, respectively. The M-O bonds (530 eV) are formed by bonding O_2_- ions with elements of a-IGZO metals such as indium, gallium, and zinc. Also, the M-OH bonds (532 eV) were referred between the metals of a-IGZO and the hydroxyl group. In addition, the oxygen vacancies (531 eV) are defects of the a-IGZO channels, which were generated by oxygen atoms and are related to the carrier concentration of a-IGZO. The oxygen gas injected during sputtering reduced the oxygen vacancies of the deposited a-IGZO film. The relative areas of oxygen vacancies in C_1_, C_2_, and C_3_ were 30.6%, 29.6%, and 27.4%, respectively, which oxygen vacancies are reduced when injected oxygen gas was increased. The oxygen vacancy is known to have a significant effect on the change of electrical characteristics in a-IGZO thin firm TFTs [50]. Adequate oxygen vacancies increase the conductivity of a-IGZO by generating free electrons [51]. In addition, the M-OH bond of a-IGZO was decreased as the oxygen flow rate increased [52]. The relative area of M-OH bond in C_1_, C_2_, and C_3_ were 9.5%, 9%, and 4.95%, respectively. The M-OH bond acts as a deep-level trap that prevents charge transport in the a-IGZO, inducing the operation of the unstable TFT [53]. As a result, the optimized C_1_ TFT has improved electrical characteristics due to adequate oxygen vacancies and reduced M-OH bonding.

Moreover, the changes in the optical bandgap of a-IGZO with oxygen gas flow were investigated. Figure 5d–f shows the optical bandgap of a-IGZO calculated by the Tauc plot method using UV–visible absorption data. The a-IGZO in the C_1_ condition without oxygen injection showed the smallest optical bandgap of 3.63 eV. On the other hand, the optical bandgaps of C_2_ and C_3_ increased by oxygen injection are 3.81 eV and 4.34 eV, respectively. As a result, the optical bandgap of a-IGZO was increased as the injected oxygen gas was increased.

## 4. Conclusions

In summary, the electrical characteristics of oxygen flow rate injection were investigated. As a result, when oxygen flow rate injection was not performed, the best electrical characteristics such as mobility, V_to_, and on/off ratio were shown. The decreased oxygen vacancy in a-IGZO caused by an increased oxygen gas flow rate results in a decreased conductivity. The C_1_ TFT without oxygen gas injected has enhanced charge carrier mobility due to high oxygen vacancy. In addition, AFM and XPS analyses were performed to confirm the mechanism of the change in electrical properties due to oxygen flow injection. In the AFM analysis, the improvement of electrical properties was confirmed through wide contact with the electrode material because the C_1_ device had the greatest roughness. Finally, through XPS analysis, it was confirmed that the electrical characteristics of the C_1_ device with the highest oxygen vacancy were the best. Through this result, we believe that the development of oxide semiconductor research and process condition research will be inexhaustible.
